# (2*E*)-*N*′-[(*E*)-2-Hy­droxy­benzyl­idene]-3-phenyl­prop-2-enohydrazide

**DOI:** 10.1107/S1600536812028516

**Published:** 2012-06-30

**Authors:** Samir A. Carvalho, Edson F. da Silva, Carlos A. M. Fraga, Solange M. S. V. Wardell, James L. Wardell, Edward R. T. Tiekink

**Affiliations:** a FioCruz-Fundação Oswaldo Cruz, Instituto de Tecnologia em Fármacos-Farmanguinhos, Rua Sizenando Nabuco, 100, Manguinhos, 21041-250 Rio de Janeiro, RJ, Brazil; b Programa de Pós-Graduação em Química, Instituto de Química, Universidade Federal do Rio de Janeiro, 21949-900 Rio de Janeiro, RJ, Brazil; c Laboratório de Avaliação e Síntese de Substâncias Bioativas, Faculdade de Farmácia, Universidade Federal do Rio de Janeiro, PO Box 68023, 21941-902 Rio de Janeiro, RJ, Brazil; d CHEMSOL, 1 Harcourt Road, Aberdeen AB15 5NY, Scotland; e Centro de Desenvolvimento Tecnológico em Saúde (CDTS), Fundação Oswaldo Cruz (FIOCRUZ), Casa Amarela, Campus de Manguinhos, Av. Brasil 4365, 21040-900 Rio de Janeiro, RJ, Brazil; f Department of Chemistry, University of Malaya, 50603 Kuala Lumpur, Malaysia

## Abstract

In the non-planar title compound, C_16_H_14_N_2_O_2_, the dihedral angle between the phenyl rings is 16.67 (8)°. An *E* conformation is found for each of the imine [1.286 (2) Å] and ethyl­ene [1.335 (2) Å] bonds. The amide O and H atoms are *anti*, and an intra­molecular hy­droxy O—H⋯N hydrogen bond is noted. The formation of N—H⋯O(hy­droxy) hydrogen bonds in the crystal packing leads to helical chains along the *b* axis. Supra­molecular layers in the *ab* plane are formed as the chains are linked by C—H⋯O inter­actions.

## Related literature

For background to the biological activity of compounds with the *N*-acyl­hydrazone framework, (*E*)-cinnamoylhydrazone derivatives, and related structures, see: Carvalho *et al.* (2012*a* ▶). For the synthesis, see: Carvalho *et al.* (2012*b* ▶). For background to the data collection at the National Crystallographic Service, see: Coles & Gale (2012 ▶).
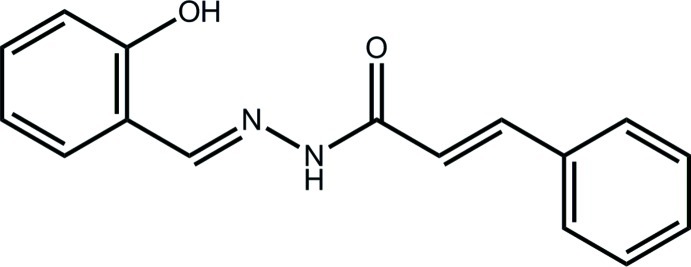


## Experimental

### 

#### Crystal data

C_16_H_14_N_2_O_2_
*M* *_r_* = 266.29
Orthorhombic, 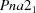

*a* = 24.2707 (17) Å
*b* = 5.1322 (2) Å
*c* = 10.5192 (4) Å
*V* = 1310.29 (12) Å^3^
*Z* = 4
Mo *K*α radiation
μ = 0.09 mm^−1^
*T* = 100 K
0.19 × 0.09 × 0.03 mm

#### Data collection

Rigaku Saturn724+ diffractometer
Absorption correction: multi-scan (*CrystalClear*; Rigaku, 2011 ▶) *T*_min_ = 0.878, *T*_max_ = 1.000
5871 measured reflections
1575 independent reflections
1504 reflections with *I* > 2σ(*I*)
*R*_int_ = 0.022

#### Refinement

*R*[*F* ^2^ > 2σ(*F* ^2^)] = 0.028
*wR*(*F* ^2^) = 0.075
*S* = 0.93
1575 reflections
181 parameters
1 restraint
H atoms treated by a mixture of independent and constrained refinement
Δρ_max_ = 0.20 e Å^−3^
Δρ_min_ = −0.15 e Å^−3^

### 

Data collection: *CrystalClear* (Rigaku, 2011 ▶); cell refinement: *CrystalClear*; data reduction: *CrystalClear*; program(s) used to solve structure: *SHELXS97* (Sheldrick, 2008 ▶); program(s) used to refine structure: *SHELXL97* (Sheldrick, 2008 ▶); molecular graphics: *ORTEP-3 for Windows* (Farrugia, 1997 ▶) and *DIAMOND* (Brandenburg, 2006 ▶); software used to prepare material for publication: *publCIF* (Westrip, 2010 ▶).

## Supplementary Material

Crystal structure: contains datablock(s) global, I. DOI: 10.1107/S1600536812028516/hb6860sup1.cif

Structure factors: contains datablock(s) I. DOI: 10.1107/S1600536812028516/hb6860Isup2.hkl

Supplementary material file. DOI: 10.1107/S1600536812028516/hb6860Isup3.cml

Additional supplementary materials:  crystallographic information; 3D view; checkCIF report

## Figures and Tables

**Table 1:** Hydrogen-bond geometry (Å, °)

*D*—H⋯*A*	*D*—H	H⋯*A*	*D*⋯*A*	*D*—H⋯*A*
O1—H1o⋯N1	0.85 (2)	1.86 (2)	2.6080 (19)	147 (2)
N2—H2n⋯O1^i^	0.88 (1)	2.05 (1)	2.9070 (19)	165 (2)
C3—H3⋯O2^ii^	0.95	2.54	3.215 (2)	128
C7—H7⋯O2^ii^	0.95	2.47	3.174 (2)	131

Symmetry codes: (i) 
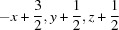
; (ii) 
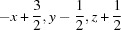
.

**Fig. 1.**
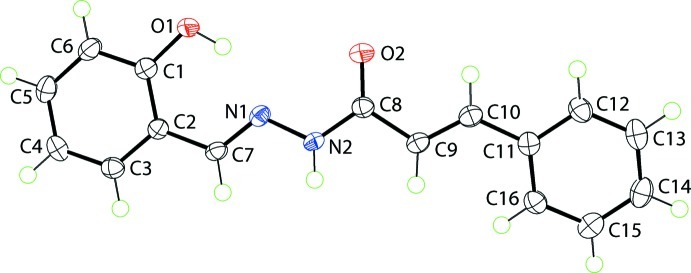
The molecular structure of (I) showing the atom-labelling scheme and displacement ellipsoids at the 70% probability level.

**Fig. 2.**
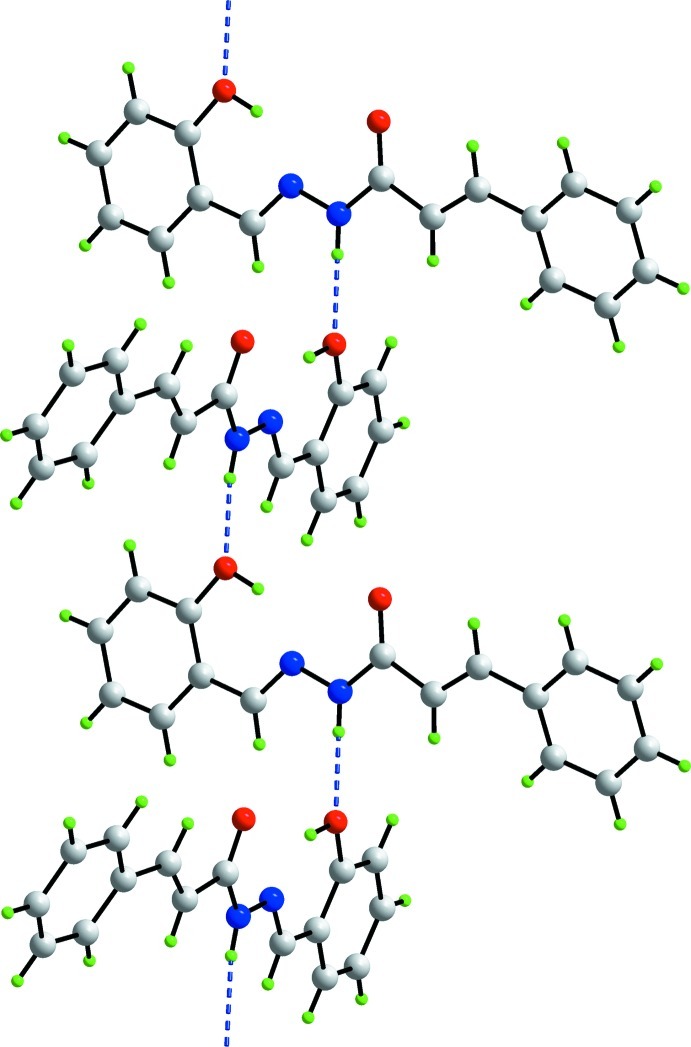
A view of the supramolecular helical chain along the b axis in (I). The N—H···O hydrogen bonds are shown as blue dashed lines.

**Fig. 3.**
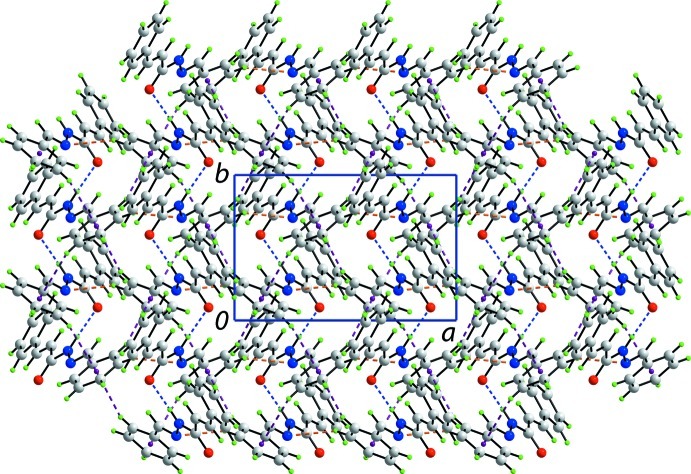
A view of the supramolecular layer in the ab plane in (I) sustained by N—H···O and interactions, shown as blue and orange dashed lines, respectively.

**Fig. 4.**
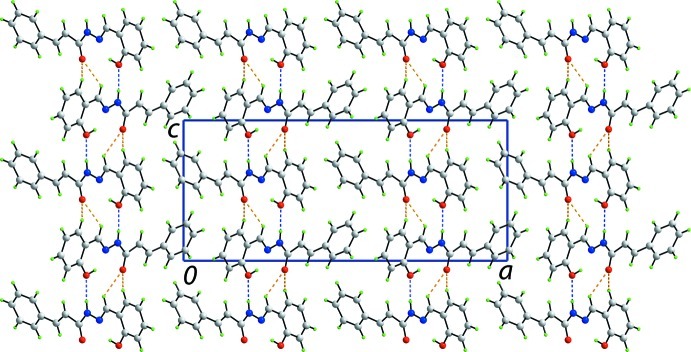
A view in projection down the b axis of the unit-cell contents for (I) showing the inter-digitation of layers. The N—H···O and C—H···O interactions are shown as blue and orange dashed lines, respectively.

## supplementary crystallographic information

### Comment

Compounds related to the title (*E*)-cinnamoylhydrazone derivative, (I), are of interest owing to their biological activities (Carvalho *et al.*, 2012*a*). For example, (I), exhibits considerable trypanocidal activity (Carvalho *et al.*, 2012*b*). Herein, the crystal structure determination of (I) is described.

In (I), Fig. 1, there is a twist in the molecule as seen in the dihedral angle between the phenyl rings of 16.67 (8)°. The greatest deviation from a planar torsion angle is found for C9—C10—C11—C16 of 8.4 (3)°. There is an intramolecular hydroxy-O1···N2 hydrogen bond. The conformation about each of the imine [N1═C7 = 1.286 (2) Å] and ethylene [C9═C10 = 1.335 (2) Å] bonds is *E*. The amide-O and –H atoms are *anti*. The molecular structure of (I) resembles that of the unsubstituted compound (Carvalho *et al.*, 2012*a*) where the dihedral angle between terminal phenyl rings is 25.48 (12)°.

The formation of N—H···O hydrogen bonds between the amide-H and hydroxyl-O leads to helical supramolecular chains along the *b* axis, Fig. 2 and Table 1. The chains are linked into a supramolecular layer in the *ab* plane by C—H···O interactions, Fig. 3 and Table 1; the layers inter-digitate along the *c* axis, Fig. 4.

### Experimental

The title compound was prepared as reported (Carvalho *et al.*, 2012*b*). The sample used in the crystallographic study was grown from its EtOH solution and intensity data was collected at the National Crystallographic Service, England (Coles & Gale, 2012).

### Refinement

The C-bound H atoms were geometrically placed (C—H = 0.95 Å) and refined as riding with *U*_iso_(H) = 1.2*U*_eq_(C). The O– and N-bound H atoms were located from a difference map and refined with the distance restraint O—H = 0.84±0.01 and N—H = 0.88±0.01 Å, and with *U*_iso_(H) = *zU*_eq_(carrier atom); *z* = 1.5 for O and *z* = 1.2 for N. In the absence of significant anomalous scattering effects, 1033 Friedel pairs were averaged in the final refinement. One reflection, *i.e.* (20 0 0) was omitted from the final refinement owing to poor agreement.

### Crystal data

**Table:**

C_16_H_14_N_2_O_2_	*F*(000) = 560
*M**_r_* = 266.29	*D*_x_ = 1.350 Mg m^−^^3^
Orthorhombic, *P**n**a*2_1_	Mo *K*α radiation, λ = 0.71073 Å
Hall symbol: P 2c -2n	Cell parameters from 5659 reflections
*a* = 24.2707 (17) Å	θ = 3.4–27.5°
*b* = 5.1322 (2) Å	µ = 0.09 mm^−^^1^
*c* = 10.5192 (4) Å	*T* = 100 K
*V* = 1310.29 (12) Å^3^	Plate, colourless
*Z* = 4	0.19 × 0.09 × 0.03 mm

### Data collection

**Table:**

Rigaku Saturn724+ diffractometer	1575 independent reflections
Radiation source: Rotating Anode	1504 reflections with *I* > 2σ(*I*)
Confocal monochromator	*R*_int_ = 0.022
Detector resolution: 28.5714 pixels mm^-1^	θ_max_ = 27.5°, θ_min_ = 3.4°
profile data from ω–scans	*h* = −30→31
Absorption correction: multi-scan (*CrystalClear*; Rigaku, 2011)	*k* = −6→6
*T*_min_ = 0.878, *T*_max_ = 1.000	*l* = −13→13
5871 measured reflections	

### Refinement

**Table:**

Refinement on *F*^2^	Secondary atom site location: difference Fourier map
Least-squares matrix: full	Hydrogen site location: inferred from neighbouring sites
*R*[*F*^2^ > 2σ(*F*^2^)] = 0.028	H atoms treated by a mixture of independent and constrained refinement
*wR*(*F*^2^) = 0.075	*w* = 1/[σ^2^(*F*_o_^2^) + (0.0497*P*)^2^ + 0.3579*P*] where *P* = (*F*_o_^2^ + 2*F*_c_^2^)/3
*S* = 0.93	(Δ/σ)_max_ = 0.001
1575 reflections	Δρ_max_ = 0.20 e Å^−^^3^
181 parameters	Δρ_min_ = −0.15 e Å^−^^3^
1 restraint	Absolute structure: nd
Primary atom site location: structure-invariant direct methods	

### Special details

**Table:**

Geometry. All s.u.'s (except the s.u. in the dihedral angle between two l.s. planes) are estimated using the full covariance matrix. The cell s.u.'s are taken into account individually in the estimation of s.u.'s in distances, angles and torsion angles; correlations between s.u.'s in cell parameters are only used when they are defined by crystal symmetry. An approximate (isotropic) treatment of cell s.u.'s is used for estimating s.u.'s involving l.s. planes.
Refinement. Refinement of *F*^2^ against ALL reflections. The weighted *R*-factor *wR* and goodness of fit *S* are based on *F*^2^, conventional *R*-factors *R* are based on *F*, with *F* set to zero for negative *F*^2^. The threshold expression of *F*^2^ > 2σ(*F*^2^) is used only for calculating *R*-factors(gt) *etc*. and is not relevant to the choice of reflections for refinement. *R*-factors based on *F*^2^ are statistically about twice as large as those based on *F*, and *R*- factors based on ALL data will be even larger.

### Fractional atomic coordinates and isotropic or equivalent isotropic displacement parameters (Å^2^)

**Table:**

	*x*	*y*	*z*	*U*_iso_*/*U*_eq_	
O1	0.69975 (5)	0.5777 (2)	−0.10431 (12)	0.0192 (3)	
H1O	0.7247 (7)	0.671 (4)	−0.071 (2)	0.029*	
O2	0.81273 (5)	1.1490 (3)	−0.06463 (13)	0.0232 (3)	
N1	0.75962 (6)	0.7782 (3)	0.07685 (14)	0.0150 (3)	
N2	0.79692 (6)	0.9517 (3)	0.12637 (14)	0.0158 (3)	
H2N	0.7997 (9)	0.959 (4)	0.2096 (10)	0.019*	
C1	0.67996 (7)	0.4128 (3)	−0.01381 (17)	0.0157 (3)	
C2	0.69722 (7)	0.4288 (3)	0.11420 (17)	0.0140 (3)	
C3	0.67560 (7)	0.2498 (3)	0.20137 (17)	0.0171 (4)	
H3	0.6869	0.2577	0.2877	0.021*	
C4	0.63806 (7)	0.0613 (3)	0.16428 (19)	0.0187 (4)	
H4	0.6241	−0.0602	0.2244	0.022*	
C5	0.62097 (7)	0.0517 (3)	0.03833 (19)	0.0201 (4)	
H5	0.5947	−0.0753	0.0128	0.024*	
C6	0.64170 (7)	0.2249 (3)	−0.05053 (18)	0.0197 (4)	
H6	0.6298	0.2157	−0.1365	0.024*	
C7	0.73698 (7)	0.6215 (3)	0.15666 (17)	0.0150 (3)	
H7	0.7463	0.6319	0.2442	0.018*	
C8	0.82227 (7)	1.1293 (3)	0.04921 (17)	0.0156 (3)	
C9	0.86435 (7)	1.2853 (3)	0.11785 (17)	0.0161 (3)	
H9	0.8711	1.2542	0.2055	0.019*	
C10	0.89272 (7)	1.4693 (3)	0.05648 (17)	0.0170 (3)	
H10	0.8815	1.5065	−0.0281	0.020*	
C11	0.93932 (7)	1.6197 (3)	0.10555 (17)	0.0159 (3)	
C12	0.96062 (7)	1.8241 (3)	0.03252 (19)	0.0203 (4)	
H12	0.9442	1.8652	−0.0469	0.024*	
C13	1.00558 (8)	1.9677 (4)	0.0748 (2)	0.0226 (4)	
H13	1.0196	2.1061	0.0242	0.027*	
C14	1.03004 (7)	1.9097 (4)	0.19034 (19)	0.0225 (4)	
H14	1.0606	2.0087	0.2193	0.027*	
C15	1.00967 (7)	1.7064 (4)	0.26359 (18)	0.0210 (4)	
H15	1.0266	1.6658	0.3426	0.025*	
C16	0.96482 (7)	1.5624 (3)	0.22228 (18)	0.0177 (3)	
H16	0.9512	1.4238	0.2732	0.021*	

### Atomic displacement parameters (Å^2^)

**Table:**

	*U*^11^	*U*^22^	*U*^33^	*U*^12^	*U*^13^	*U*^23^
O1	0.0245 (6)	0.0213 (6)	0.0118 (6)	−0.0067 (5)	−0.0012 (5)	−0.0003 (5)
O2	0.0293 (7)	0.0274 (7)	0.0130 (6)	−0.0075 (5)	−0.0028 (6)	−0.0001 (6)
N1	0.0147 (6)	0.0154 (6)	0.0149 (6)	−0.0003 (5)	−0.0009 (5)	−0.0031 (6)
N2	0.0175 (7)	0.0183 (7)	0.0115 (7)	−0.0035 (5)	−0.0015 (6)	−0.0028 (6)
C1	0.0148 (7)	0.0167 (7)	0.0154 (8)	0.0021 (6)	0.0008 (6)	−0.0019 (7)
C2	0.0136 (7)	0.0144 (7)	0.0141 (8)	0.0011 (6)	0.0011 (6)	−0.0023 (7)
C3	0.0170 (8)	0.0190 (8)	0.0154 (9)	0.0017 (6)	0.0011 (7)	−0.0009 (7)
C4	0.0189 (8)	0.0156 (8)	0.0215 (9)	−0.0003 (6)	0.0054 (7)	0.0021 (7)
C5	0.0175 (8)	0.0179 (8)	0.0248 (9)	−0.0029 (6)	0.0001 (7)	−0.0049 (7)
C6	0.0189 (8)	0.0225 (8)	0.0177 (8)	−0.0014 (7)	−0.0032 (7)	−0.0041 (8)
C7	0.0150 (7)	0.0182 (8)	0.0118 (7)	0.0005 (6)	−0.0007 (6)	−0.0029 (7)
C8	0.0159 (7)	0.0166 (8)	0.0143 (8)	0.0010 (6)	0.0009 (6)	−0.0013 (7)
C9	0.0167 (7)	0.0174 (8)	0.0141 (7)	0.0008 (6)	−0.0010 (6)	−0.0024 (7)
C10	0.0175 (8)	0.0176 (8)	0.0158 (8)	0.0015 (6)	−0.0009 (7)	−0.0018 (7)
C11	0.0148 (7)	0.0148 (7)	0.0180 (8)	0.0017 (6)	0.0028 (7)	−0.0019 (7)
C12	0.0212 (8)	0.0180 (8)	0.0217 (9)	0.0004 (6)	0.0004 (7)	0.0039 (7)
C13	0.0207 (9)	0.0160 (8)	0.0312 (10)	−0.0016 (6)	0.0038 (8)	0.0015 (8)
C14	0.0175 (8)	0.0189 (8)	0.0311 (11)	−0.0004 (7)	0.0009 (7)	−0.0060 (8)
C15	0.0178 (8)	0.0230 (9)	0.0223 (9)	0.0025 (7)	−0.0015 (7)	−0.0046 (8)
C16	0.0173 (7)	0.0174 (8)	0.0182 (8)	0.0010 (6)	0.0030 (7)	−0.0001 (7)

### Geometric parameters (Å, º)

**Table:**

O1—C1	1.361 (2)	C7—H7	0.9500
O1—H1O	0.848 (10)	C8—C9	1.485 (2)
O2—C8	1.224 (2)	C9—C10	1.335 (2)
N1—C7	1.286 (2)	C9—H9	0.9500
N1—N2	1.3727 (19)	C10—C11	1.463 (2)
N2—C8	1.367 (2)	C10—H10	0.9500
N2—H2N	0.879 (10)	C11—C12	1.399 (2)
C1—C6	1.393 (2)	C11—C16	1.406 (2)
C1—C2	1.413 (3)	C12—C13	1.390 (3)
C2—C3	1.400 (2)	C12—H12	0.9500
C2—C7	1.452 (2)	C13—C14	1.385 (3)
C3—C4	1.385 (2)	C13—H13	0.9500
C3—H3	0.9500	C14—C15	1.388 (3)
C4—C5	1.389 (3)	C14—H14	0.9500
C4—H4	0.9500	C15—C16	1.386 (2)
C5—C6	1.385 (3)	C15—H15	0.9500
C5—H5	0.9500	C16—H16	0.9500
C6—H6	0.9500		
			
C1—O1—H1O	108.1 (18)	O2—C8—C9	124.15 (16)
C7—N1—N2	116.09 (15)	N2—C8—C9	112.36 (15)
C8—N2—N1	120.28 (15)	C10—C9—C8	120.06 (16)
C8—N2—H2N	122.0 (15)	C10—C9—H9	120.0
N1—N2—H2N	117.1 (15)	C8—C9—H9	120.0
O1—C1—C6	118.13 (17)	C9—C10—C11	126.95 (16)
O1—C1—C2	121.73 (15)	C9—C10—H10	116.5
C6—C1—C2	120.14 (17)	C11—C10—H10	116.5
C3—C2—C1	118.36 (15)	C12—C11—C16	118.26 (16)
C3—C2—C7	119.64 (16)	C12—C11—C10	119.17 (16)
C1—C2—C7	121.99 (16)	C16—C11—C10	122.55 (16)
C4—C3—C2	121.36 (17)	C13—C12—C11	120.80 (18)
C4—C3—H3	119.3	C13—C12—H12	119.6
C2—C3—H3	119.3	C11—C12—H12	119.6
C3—C4—C5	119.33 (17)	C14—C13—C12	120.23 (18)
C3—C4—H4	120.3	C14—C13—H13	119.9
C5—C4—H4	120.3	C12—C13—H13	119.9
C6—C5—C4	120.84 (17)	C13—C14—C15	119.73 (17)
C6—C5—H5	119.6	C13—C14—H14	120.1
C4—C5—H5	119.6	C15—C14—H14	120.1
C5—C6—C1	119.96 (17)	C16—C15—C14	120.44 (17)
C5—C6—H6	120.0	C16—C15—H15	119.8
C1—C6—H6	120.0	C14—C15—H15	119.8
N1—C7—C2	120.60 (16)	C15—C16—C11	120.54 (17)
N1—C7—H7	119.7	C15—C16—H16	119.7
C2—C7—H7	119.7	C11—C16—H16	119.7
O2—C8—N2	123.43 (16)		
			
C7—N1—N2—C8	−178.71 (15)	N1—N2—C8—O2	1.8 (3)
O1—C1—C2—C3	−179.07 (15)	N1—N2—C8—C9	−175.41 (14)
C6—C1—C2—C3	1.0 (2)	O2—C8—C9—C10	3.3 (3)
O1—C1—C2—C7	0.0 (2)	N2—C8—C9—C10	−179.52 (15)
C6—C1—C2—C7	−179.97 (16)	C8—C9—C10—C11	−172.84 (16)
C1—C2—C3—C4	−0.2 (2)	C9—C10—C11—C12	−173.45 (17)
C7—C2—C3—C4	−179.29 (15)	C9—C10—C11—C16	8.4 (3)
C2—C3—C4—C5	−0.8 (3)	C16—C11—C12—C13	−0.4 (3)
C3—C4—C5—C6	1.1 (3)	C10—C11—C12—C13	−178.64 (16)
C4—C5—C6—C1	−0.4 (3)	C11—C12—C13—C14	0.1 (3)
O1—C1—C6—C5	179.34 (15)	C12—C13—C14—C15	0.4 (3)
C2—C1—C6—C5	−0.7 (3)	C13—C14—C15—C16	−0.4 (3)
N2—N1—C7—C2	−179.55 (14)	C14—C15—C16—C11	0.1 (3)
C3—C2—C7—N1	176.11 (15)	C12—C11—C16—C15	0.3 (2)
C1—C2—C7—N1	−2.9 (2)	C10—C11—C16—C15	178.50 (16)

### Hydrogen-bond geometry (Å, º)

**Table:**

*D*—H···*A*	*D*—H	H···*A*	*D*···*A*	*D*—H···*A*
O1—H1o···N1	0.85 (2)	1.86 (2)	2.6080 (19)	147 (2)
N2—H2n···O1^i^	0.88 (1)	2.05 (1)	2.9070 (19)	165 (2)
C3—H3···O2^ii^	0.95	2.54	3.215 (2)	128
C7—H7···O2^ii^	0.95	2.47	3.174 (2)	131

Symmetry codes: (i) −*x*+3/2, *y*+1/2, *z*+1/2; (ii) −*x*+3/2, *y*−1/2, *z*+1/2.
